# An Essay on the Nature and Treatment of Erysipelas

**Published:** 1861

**Authors:** James S. Whitmire

**Affiliations:** Metamora, Ills.


CHICAGO

MEDICAL JOURNAL.

VOL. IV.] JUNE & JULY, 1861. [NOS. 6 & 7.

### AN ESSAY

### ON THE NATURE AND TREATMENT OF ERYSIPELAS.

By JAMES S. WHITMIRE, M. D., of Metamora, Ills.

Erysipelas is a disease that prevails in every country and climate on the face of the earth; and neither age, sex, nor condition is exempt from its influence in one or another of its various forms. It, therefore, being a dissease so general, and one, too, that baffles the skill of the most judicious and erudite in the profession, on account of its stubborn, erratic, and varied character, according to circumstances, it becomes us as members of a noble profession, and ministers of mercy to the afflicted of our common humanity, who are seeking after knowledge, to each do his part in the investigation of the different theories and treatment of every disease that comes within the purview of our observations, to compare notes and interchange opinions in regard to them that we may be the better qualified to form a correct judgment in respect to their treatment and cure. And *particularly* is this the case in relation to the disease under consideration. Because there is no disease, probably, that flesh is heir to, that is more prevalent, nor that has had a greater variety of treatment applied for its cure, and none requiring, under the different circumstances in which it prevails, application of remedial agents. It is not my province in this paper to enter minutely, into either the history, cause, nature, varieties or diagnosis of this disease:—these are discussed at length in all our standard works on theory and practice, and are sufficiently well understood; so that there is a general understanding in regard to them, but the agreement in respect to its treatment, seems to be not quite so amicably settled. I will, therefore, only advert to so much of the generally received opinions as to its nature, as to go to what would most naturally make up the general indications of treatment, and then what remedial agents, in my opinion, would be most likely to fulfill those indications. While I am free to admit that many of us spend too much of our time in fruitlessly seeking after specifics and infallible remedies for particular diseases, and that in consequence we are too prone to fall into a routine practice and treat names and not diseases as they occur; yet, there is a general plan of treatment in all diseases, without regard to particular circumstances, that must be adhered to in order to bring about certain ends whether they be accomplished by one or another means; and in no disease does this principle hold good more certainly than in Erysipelas. As proof of this statement, you only have to refer to our standard authors and witness the reliance that each places on his particular method of treatment, under the different circumstances, and yet all converging to the same end by different means, and hence, probably, arises the great variety of treatment in this disease; than which no better proof could be adduced of the general inefficiency of all the means heretofore used, is this same diversity of opinion. And, yet, all the different means used, probably, under the different surroundings, influences or peculiarities, may have been the best that could be selected under the circumstances, hence the necessity of close observation and a discriminating judgment in the professional man. It has fallen to my lot in a country practice, of fifteen years, to have seen this disease in its various forms and under as various circmstances as almost any other practitioner of my age, and though my patients have universally recovered, yet I have never been entirely satisfied till within the last few years that the usual remedies used are not the best we have at our command. Since that time, I have viewed erysipelas as not only a constitutional disease, but dependent, in some degree, upon epidemic influence, and an animal poison for its propagation. In all instances in localities where erysipelas is epidemic, the whole community becomes predisposed to the disease from these influences, and the infection on account of the predisposition becomes doubly sure of propagation. And in all instances where there is a strong predisposition, or in idiosyncratic cases where the epidemic influence prevails, the disease may be developed without any contact whatever. And even though it may be developed without contact, it is none the less a disease of infection by contact, but not, probably, to the same extent as many others of our acknowledged contagious diseases. On the other hand it is argued against its being contagious, that it is not self-limited, nor does it fortify the system against a second attack, which is the case with other diseases of this class, and therefore should be thrown out of the list of contagions. But this would give but little scope to our investigations, and little encouragement to the free expression of our opinions, and narrow down and limit our observations to that which is acknowledged; and thus effectually close the door to all improvement. Because the first contagious diseases that were known to the profession were self-limited, and fortified the system against a second attack, is no evidence, to my mind, that it may not yet be found that other diseases may be communicable by contact or proximity and yet not partake of the other two characteristics.

I have come to the conclusion, from observation, without going into particulars, that we have many infections, or diseases that may become so under favorable circumstances that are not now recognized; and indeed, not easily recognizable, from the fact that they do not present all the characteristics of our acknowledged contagious diseases. So far, however, as this matter of contagion is concerned, it matters but little, either here or there, in regard to the disease under consideration, and I have only given my own convictions, without going into the particulars or data, from which they were formed; but such was their force at the time that I have become a thorough convert to the doctrine, and simply state the fact that investigation may be elicited by the profession. Without any reference to this hypothesis, however, there are every day occurrences to all of us where this disease is developed, apparently without any cause whatever. Exposure to cold or extreme vicissitudes of temperature—irregularity of diet—unwholesome food, creating a vitiated condition of the system—violent emotions, etc., etc., are all supposed to be frequent exciting causes of it. Many cases, too, that cannot be distinguished either in appearance, course or termination from idiopathic erysipelas, evidently result from injury. And because it may be developed by so great a variety of existing causes, where there is no apparent contagion, is no evidence to my mind, that after it is once produced, from whatever cause, the disease may not, of itself, and in itself generate the property or principle of self-propagation. All nature around us, proclaim this principle as a great fundamental truth; and may it not hold good, also, in the kingdom of disease? There are four varieties of this disease as recognized by authors—the simple or cutaneous, where the inflammatory action is superficial and confined to the skin and its membranes. The Phlegmonous, where inflammation of the skin is also present, and involving, also, the subscutaneous tissues, and not unfrequently dipping down into the cellular tissue covering the muscles. The Œdematous, which is most likely to attack patients of a cachectic habit, and in whom there is a tendency to dropsical infiltration of the cellular tissue—hence the lower limbs are the most liable to this form of the disease, and in this form neither the heat nor redness is so great as in the former varieties, but the pitting on pressure and a doughy feel of the parts are two of the distinguishing characteristics.—The Gangrenous, may be either the original form of the disease dependent on a depraved condition of the system, or the malignancy of the cause that produced it, or, it may result from the excessive violence of the inflammation in the former varieties of the disease. Assuming, then, that this is a general disease, and that it is generally dependent on an animal poison for its existence and propagation, we are then left to consider the following and only proposition in regard to its rational and therefore successful treatment--to wit:—Have we in the Materia Medica an agent, the therapeutic agency of which will at the same time destroy, or neutralize the animal poison, and give tone and vigor to the debilitated capillaries of the affected part? If we have, then, indeed would the malignancy or virulence of this sometimes terrible scourge be mitigated and consequently its danger materially lessened.—The tendency, as I understand it, in every form of this disease is debility of the capillaries and consequent swelling from atonic congestion of these vessels rendering them incapable of carrying on the healthy circulation of the blood through the diseased part, and hence the peculiar inflammation, heat, swelling and infiltration of serum into the cellular tissue of the diseased part and its immediate vicinity. And though erysipelas is known and designated in varieties or different forms, yet the disease is the same, to all intents and purposes, and under any and all circumstances; the difference being simply in degree, and the degree being modified by circumstances such as constitutional predisposition, idiosyncracy, or the virulence of the original cause. This disease, therefore, being the same under all circumstances we would most naturally conclude that a sameness in the indications, so far as the general treatment is concerned would necessarily be the most rational course to be pursued, provided we have a remedy, or remedies, the curative agency of which is calculated to moderate or immediately overcome the disease by removing the cause, and give nature a clear field to complete the cure.—This agent, in my opinion, we have found in Iodine and its preparations, it acting, not only, as an antidote to the poison, through the medium of the blood; but, also, as an alterative in rousing and changing the secretions, and as a tonic to the capillary system of the vessels, thereby instituting a curative process in the diseased parts by promoting a healthy action.—My method of using this remedy can, probably, be stated in no way more satisfactorily than by giving the history and treatment of a case of Phlegmonous Erysipelas that has within a short period been under my care, which is simply a fair comparative representation of many others of the same nature that have occurred in my practice since 1856.

Mch. 4, 1861, I was called to see Thos. Beal, aged 40, a farmer, was taken ill about four days previously with what he considered at the time a common cold. Had violent pains in the head, back and limbs, with occasional chilly sensations and flushes of fever for three consecutive days. Twenty-four hours before I was called, he could not bear to have the bedclothes moved on account of the slightest contact of air throwing him into a violent chill, notwithstanding there was great heat of the skin and thirst during the whole time. The night previously he was quite deranged, so that the family became alarmed and sent for me. I found him as just stated—pulse 120 per minute and full—skin hot—great thirst—no appetite and occasional wandering delirium. I gave him 15 gr. of calomel to be followed in three hours with an infusion of salts and senna, to be given until thorough purgation was produced. The local affection had commenced during the night on the left side of the nose, at first by a mere speck, and in ten hours it had extended to the eye and half of the cheek on the same side. After purgation he was to take the following prescription:

℞. Quin. Sulph. . . . . . . . . . . . ℈ I.

Camp. Gum Pulv., . . . . . . . . . . ℈ I.

Potass. Chlor. . . . . . . . . . . . ʒ I.

Ipec. Pulv. . . . . . . . . . . . . . gr. V.

Verat. virid. Pulv. . . . . . . . . . . gr. X.

Morph. Sulp. . . . . . . . . . . . . gr. 1.

Misce—Fiant—Pulveres . . . . . . . . X.

One to be taken every three hours. Between the powders I gave the following mixture in doses of one table-spoonful, in a common tumbler of water.

10 ℞. Potass. Iod. . . . . . . . . . . ʒ II.

Verat. virid. Tinct. . . . . . . . . gr. XXX.

Spts. Nit. Dulc. . . . . . . . . . . . f ℥ I.

Aquæ, . . . . . . . . . . . . . . f ℥ III.

The local treatment consisted of an Iodine Ointment prepared in the following manner.

℞ Iod. . . . . . . . . . . . . . . gr. XXX.

Potass Iod. . . . . . . . . . . . . . gr. X.

Alcohol. . . . . . . . . . . . . . . f. ℥ Ss.

Rubbed together in a porcelain mortar, till the Iodine is thoroughly dissolved. Then add Ol. Ricini f. ʒ III Ss. and triturate till it is thoroughly mixed. Bottle and it is ready for use. With this ointment I keep the diseased surface and two or three inches beyond, well lubricated by three, four, or five applications per day, if required to keep it soft. And, to digress a moment, this is one of the most beautiful as well as the most convenient preparations of Iodine Ointment that I have ever seen, and may be used in any case where the officinal preparation is indicated. March 5th. The inflammation had extended over both cheeks, closing both eyes, and down on the neck nearly to the clavicles and sternum. Pulse 100—full but soft—felt easier and rested better. Gave a dose of Ol. Ricini, and continued treatment adding gttXV of the Ferri. Mur. Tinct. to each dose of the mixture. March 6th. Found my patient in a mild perspiration, pulse full bowels had moved freely—during the night had free hæmorrhage from the nose—

felt still better—Disease had not extended during the last twenty-four hours. I now discontinued the verat. virid. and substituted half gr. of Opium for the Morphine and directed his powders to be taken every four hours, the mixture with the Mur. Tinct. of Iron as usual, between the powders. March 7th. Found my patient quite comfortable—the swelling had begun to recede—pulse 70 and soft—some appetite—feels cheerful—rested well during the night. Continued treatment, but lengthened the time of taking his powders to five hours—mixture between, but lessened the amount of Iod. Potas. in them, and diluted the ointment to one half its former strength, with Castor Oil. March 8th, 9th, 10th, 11th. Continued treatment during the daytime, letting him rest during the night. March 13th. Discharged my patient as convalescent and out of danger, leaving Ferri. Mur. Tinct. for him to take three times per day for several days, and ordered the surface to be kept soft with Castor Oil until the skin was sound and natural. During the whole course of treatment he was allowed to partake freely of animal broth, and when his appetite did not crave it, it was urged upon him as one of the means of cure, in the support it would give to the general system. As a change, when his appetite craved it, I allowed him milk porridge, thickened with corn starch. March 12th I was called to see Cyrus McCord, aged about 30. This patient was attacked very similarly to the former, the erysipelas, however, appearing on the right ear. In this case, the inflammation extended so rapidly that in 24 hours he was threatened with suffocation on account of the œdematous condition of glottis and the inflammation of the fauces and tonsils. For this latter complication I prescribed a gargle composed of Alum. Sulp. ʒ II. Ol. Cinnam. Tinc. f. ʒ IV. Acid. Sulp. Aromat. ʒ II. Kreosot. gtt.XXX. Syrup. Simp. or what is always at hand, common molasses, f. ℥ III Ss. This gargle seemed to give very speedy relief, and acted admirably in soothing the inflammation of the throat. Otherwise the treatment and termination of this case, was essentially the same as the former one, so that I refrain from any useless repetition. I have just now, March 29th discharged the third case that has occurred, in my practice, since the commencement of this article. This case occurred in the person of Mrs. Lamb, a German woman, aged about 50 years. The inflammation in this case commenced on the left fore-arm and was very painful, though the patient had no fever. The disease kept spreading for about five days—the surface was vesicated, and the swelling enormous at the end of that time. It now, however, began to recede, and in four or five days longer my patient was discharged. The treatment in this case was also the same as the two former ones, only, I did not consider the veratrum viride at all indicated and it was therefore left out of the prescription. Neither did I give the Opium nor Quinine, to the same extent as in the two former cases. By the way, it may be proper here to remark that this last patient assisted in taking care of the first one mentioned, and in *dressing his face with the Ointment during his illness.* There is no doubt but in every individual case there may circumstances arise or complications exist, that would cause me to vary my treatment accordingly, in-as-much as every exigency that may arise must be promptly met with its appropriate remedies; but the general indications are always the same; and in *Iodide of Potassium* and the Iodine Ointment with the necessary auxiliaries, we have a certain, safe and speedy remedy. Since I have adopted this method of treatment, erysipelas, in my neighborhood, has lost much of its terror, and I speak to my patients, of their speedy recovery, as confidently as I do of interrupting a paroxysm of intermittent with the Sulphate of Quinine. I have given this remedy in doses of 20 grs. every two hours without ever having witnessed any of the poisonous effects spoken of by authors; but the quantity of water with which I invariably dilute it, may probably prevent any gastric disturbance, that might otherwise occur, if given in a more concentrated form. Its entire innocence, therefore, in my opinion, is settled, when this precaution is observed unless perhaps in cases of idiosyncracy, which no doubt exist, as in regard to many other remedies and in these we would be compelled to resort to other means. In the case of children, that we cannot induce to drink so much water at regular intervals. I usually prepare it in the form of a nice syrup, and after they take it they will usually drink a sufficient quantity of water to prevent any evil consequences. I have used it in this manner with children six months old and upwards and have never observed any evil consequences from its administration, and have therefore settled down into the idea that it is one of our most agreeable, mild, and effectual remedies, and confidently ask the profession to give it a fair and impartial trial, and they will not be disappointed in its results, and they will be enabled in an ecstacy of delight to cry out with me, *Eureka! Eureka!*

